# A Distributed Space Target Constellation Task Planning Method Based on Adaptive Genetic Algorithm

**DOI:** 10.3390/s25175485

**Published:** 2025-09-03

**Authors:** Qinying Hu, Jing Guo, Desheng Liu

**Affiliations:** 1 School of Space Information, Space Engineering University, Beijing 101416, China; huqinying_7@outlook.com (Q.H.); gquiet@163.com (J.G.); 2 National Key Laboratory of Space Target Awareness, Beijing 101416, China

**Keywords:** space target surveillance, task planning, distributed algorithm, adaptive genetic algorithm

## Abstract

This study proposes a task planning approach for a distributed constellation dedicated to space target monitoring, grounded in an adaptive genetic algorithm. The approach is designed to address challenges such as the growing number of space targets and the complex constraints inherent in space target monitoring activities. After reviewing the research progress of distributed satellite task planning and adaptive genetic algorithms, a distributed task model featuring master-slave satellites was developed. This model integrates multi-constraint modeling and aims to optimize key performance indicators: task yield rate, task completion rate, resource utilization rate, and load balancing. To enhance the approach, the contract net algorithm is fused with the adaptive genetic algorithm: Firstly, in the tendering phase, centralized tendering is adopted to reduce communication overhead; Secondly, in the bidding phase, improved genetic mechanisms (e.g., dynamic reverse adjustment of crossover and mutation probabilities) and a dynamic population strategy are employed to generate task allocation schemes; Thirdly, in the bid evaluation and winning phase, differentiated strategies are applied to non-repetitive and repetitive tasks. Simulation validation shows that this approach can complete 80% of space target monitoring tasks, balance satellite loads effectively, and manage space target catalogs efficiently.

## 1. Introduction

As science and technology advance swiftly and human space exploration accelerates, space has become a core field for national strategic competition and security assurance [1]. In recent years, the launch process of various satellites has continued to accelerate, and coupled with the continuous rise in the number of space debris, the near-Earth space environment has become increasingly crowded, while the overall complexity of the space environment has continued to increase. Such trends have not only increased the difficulty of spacecraft access to and from space, but also posed severe challenges to traditional ground-based observation systems, resulting in the inability to achieve real-time protection for space asset security. Against this backdrop, launching space-based target surveillance satellites and constructing space-based target surveillance constellations have become an important approach to safeguarding space asset security. Space-based space target surveillance platforms are usually equipped with visible light payloads. To safeguard space assets and aid in managing space traffic, these systems are capable of passively sensing sunlight bounced off space targets, enabling the acquisition of immediate attitude, orbit data, and physical attributes of these targets. Additionally, by employing dynamic data analysis for forecasting the movement patterns of targets, they facilitate the identification, collection, and monitoring of space targets, offering crucial data assistance in monitoring the status of space targets, early alerts for collision risks, and managing health.

Compared with traditional Earth observation tasks, space-based space target surveillance tasks face more complex technical challenges: a vast array of space targets with distinct orbital patterns; a complicated interplay of movement between space-based systems and their targets; and the necessity to meet various limitations, including imaging and data transmission time windows. Such elements contribute to a growing conflict between scarce internal resources and varied requirements for tasks, leading to regular scarcities in resources. Within this framework, the challenge lies in optimizing the use of on-orbit satellite resources and achieving effective cooperation among task distribution across multiple satellites, planning for single-satellite tasks, and scheduling between satellites.

Currently, multi-satellite collaborative architectures are mainly divided into centralized and distributed types. The centralized collaborative architecture can solve and optimize task planning problems from a global perspective, but in practical applications, it has limitations such as high solution complexity, weak robustness, and insufficient scalability. However, space target surveillance tasks are characterized by high complexity, strong dynamics, and strict requirements for fault tolerance and reliability. Centralized systems are no longer capable of fulfilling the requirements for task efficiency and promptness. The distributed collaborative architecture gives full play to the intelligent and autonomous capabilities of each satellite. The system distributes computational demands uniformly across each node, facilitates worldwide information exchange and the negotiation and distribution of tasks via communication nodes, and ensures uniform global task management at these nodes for consistency. The design of this system not only boosts the flexibility and resilience of multi-satellite systems in changing conditions but also markedly elevates their dependability due to its decentralized architecture.

In satellite task planning, genetic algorithms are extensively employed and have risen to prominence as a key method, thanks to their robust global optimization potential. Nonetheless, conventional genetic algorithms face issues like susceptibility to local optimums and sensitivity to changes in parameters. Considering the complex and ever-changing nature of monitoring space targets, altering evolutionary factors to achieve a balance between convergence speed and optimization precision not only rapidly leads to improved outcomes but also circumvents the issue of local optimal traps due to static parameters, proving to be an efficient solution.

In summary, based on an in-depth analysis of the characteristics of space target surveillance tasks and clarification of the core needs of task planning, this paper establishes a distributed task planning model with a non-fixed master satellite and proposes a distributed task planning method based on an adaptive genetic algorithm, with the optimization objectives of maximizing task profitability rate, task completion rate, and resource utilization rate. The structure of the full text is arranged as follows: Section 2 elaborates on pertinent theories and the current state of research; Section 3 develops a model for a distributed task; Section 4 formulates a mechanism for planning and scheduling tasks in a distributed manner, utilizing an adaptive genetic algorithm; Section 5 confirms the method’s efficacy via simulation tests and performs analysis of outcomes; Section 6 summarizes the research conclusions.

## 2. Related Theories and Research

### 2.1. Distributed Satellite Task Planning

**Figure 1:**
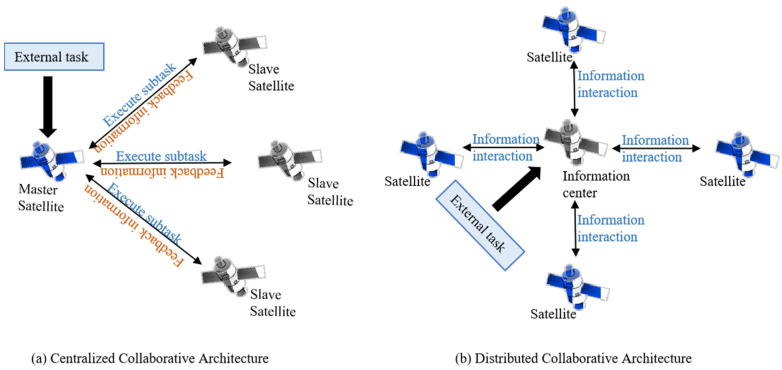
Multi-satellite collaborative architecture.

Traditional satellite task planning relies on ground stations to formulate task schemes based on preset conditions such as satellite status and mission requirements. With the increasing number of satellites and the growing dynamics of tasks, this centralized working mode consumes substantial computing resources and time. However, the continuous improvement of on-board computing capabilities has provided technical support for the autonomy of satellite task planning, making constellation collaborative autonomous task planning a technical development trend [2]. Currently, mainstream multi-satellite collaborative architectures are mainly divided into centralized collaborative architectures and distributed collaborative architectures, As shown in Figure 1.

Among them, the centralized collaborative architecture can solve and optimize task planning problems from a global perspective, but in actual planning processes, it has limitations such as high computational complexity, poor robustness, and insufficient scalability. However, space target surveillance tasks are characterized by high complexity, strong dynamics, and strict requirements for fault tolerance and reliability. The traditional centralized collaborative architecture can hardly meet the task requirements for planning quality and timeliness.

The distributed collaborative architecture, on the other hand, fully unleashes the intelligent and autonomous potential of each satellite. It evenly distributes computational loads across each node and achieves global information interaction through designated communication nodes to complete task negotiation and allocation. Meanwhile, global tasks are managed uniformly at these communication nodes to ensure consistency in the global task status. This architecture significantly enhances the adaptability and robustness of multi-satellite systems to dynamic environments, and its decentralized design further improves system reliability.

Thus, distributed architecture has gradually become a primary research direction for solving multi-satellite mission planning problems. Specifically, in their research on remote sensing satellite cluster scheduling, Skobelev et al. [3] focused on dynamic scenarios such as new task addition, task modification, and satellite or communication failure. They employed a multi-agent planning system architecture for experimental solution and systematically summarized the core advantages of the Multi-Agent system—a typical distributed architecture. Furthermore, targeting the multi-satellite mission planning problem, Zhao et al. [4] constructed a hierarchical parallel evolutionary algorithm framework based on a distributed multi-threaded two-level structure; experimental verification shows that this architecture can significantly reduce computational time consumption and effectively improve algorithm solving efficiency.

**Figure 2:**
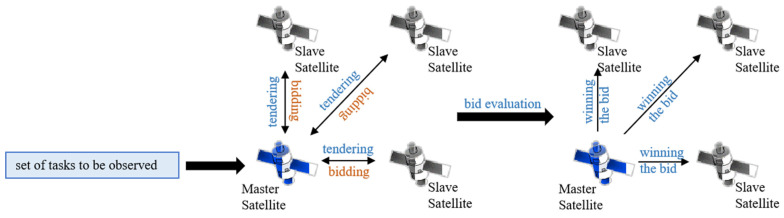
Schematic Diagram of Contract Net Algorithm Application Process.

To implement distributed architecture in multi-satellite mission planning, a variety of distributed task planning algorithms have been developed in practice. Common distributed task planning algorithms mainly include the contract net model [5], blackboard model [6], game theory [7], and market protocol methods. Among them, the contract net model is currently the most widely used and classic task planning algorithm, its application process is shown in Figure 2.

The contract net protocol (CNP) was proposed by Randall Davis and Reid G. Smith [8] in 1980 to address task allocation and resource scheduling in distributed scenarios. Its core idea is to simulate the bidding and evaluation mechanisms in human activities to achieve task planning, forming a principal-agent contractual relationship with low cost and high quality through four stages: tendering, bidding, awarding, and contracting [9]. This mechanism is characterized by strong dynamic adaptability and high task allocation efficiency, thus gaining extensive attention and application in distributed task scheduling.

To meet the rapid response requirement for marine targets, Zhang et al. [10] improved the contract net algorithm by leveraging the real-time computing capability and flexibility of satellites, enabling rapid response to emergency events. Du et al. [11] proposed a secondary allocation strategy based on task clustering, which enhances the system’s adaptability to dynamic environments and effectively improves task allocation efficiency. Cheng et al. [12]. proposed a hierarchical solution framework for distributed task scheduling: the top layer of the framework first divides tasks and satellites into corresponding clusters, and the bottom layer then allocates tasks to satellites based on the contract net protocol.

In the distributed task planning model of space target surveillance constellations, the master satellite acts as the tenderer, and the slave satellites act as the bidders. Space target surveillance tasks serve as the tendering information (released by the master satellite). The slave satellites generate a bid containing their own task planning scheme by analyzing the tendering information and their own resource capabilities and submit it to the master satellite; the master satellite evaluates each scheme according to the optimization objectives, determines the global planning scheme (i.e., contract), and distributes it to each slave satellite for execution.

The traditional contract net algorithm faces many problems in solving the task planning problem of large-scale space target surveillance constellations. Firstly, the traditional contract net algorithm adopts the “single task round-robin bidding” strategy [13], which will generate massive communication data in large-scale task scenarios. If the communication between satellites is not ideal, the cumbersome negotiation process may cause the entire bidding process to fail to be completed within the specified time, resulting in some tasks not being executed. Secondly, in the traditional contract net algorithm, when generating bidding schemes, slave satellites adopt an “insertion” strategy. If the visible time window of the later bidding task conflicts with the observation time of the planned task, the slave satellite will directly give up bidding. This way cannot adjust the generated scheme, which not only limits the utilization rate of satellite resources, but also may reduce the total task yield. Current scholars have made targeted improvements to the above two problems, mainly focusing on two directions: Huang et al. [14] combined multiple individuals into an alliance to participate in task allocation, which effectively reduced the calculation amount in the negotiation process, but may fall into local optima and ignore the existence of global optimal solutions; Jiang et al. [15]. established a task efficiency function to improve the bidding strategy to address the problems of too many bids and uneven load in the traditional contract net algorithm, and improved the reliability of task allocation by improving the basic mechanism. However, further research is still needed on the application of the contract net algorithm in solving the task planning problem of large-scale space target surveillance constellations.

### 2.2. Adaptive Genetic Algorithm

The genetic algorithm is an efficient intelligent optimization algorithm that evolved from biological knowledge such as Darwin’s theory of evolution and is based on natural selection and genetic mechanisms, simulating the law of survival of the fittest. Similar to the evolutionary process in nature, the smallest unit of operation in genetic algorithms is the gene. A set of a certain number of genes constitutes a chromosome, i.e., an individual. Each individual corresponds to a candidate feasible solution to the problem, and multiple individuals constitute a population of a generation. In each generation, the quality of individuals is evaluated by comparing the fitness value that measures the individual’s ability to adapt to the environment. Better individuals have a higher probability of being selected into the next generation. The crossover operation corresponding to gene recombination and the mutation operation corresponding to gene mutation occur with a certain probability, so as to obtain new individuals as offspring. With the evolution process, the ability of new generation individuals to adapt to the environment becomes stronger and stronger, the average fitness value of the new generation population becomes higher and higher, and the population continues to approach the optimal solution of the problem.

Genetic algorithms are widely used in satellite task planning problems and have good global optimization capabilities. Li et al. [16] designed a two-stage genetic scheduling algorithm to solve the problem of multi-satellite and multi-constraint ground measurement and control station scheduling, but the algorithm has low computational efficiency and is prone to local optimal solutions; Yuan et al. [17] improved the crossover and mutation processes of traditional genetic algorithms to solve the problem of measurement and control resource scheduling for low-orbit satellite communication constellations. The improved genetic algorithm has high efficiency and can stably obtain large revenue values. Wolfe et al. [18] improved the population evolution process of the genetic algorithm (GA) when solving the imaging satellite scheduling problem, effectively accelerating the search speed of the optimal solution; Sun et al. [19] proposed an improved genetic algorithm to solve the two-stage problem of multi-satellite mission resource matching and single-satellite mission scheduling, which enhanced the effectiveness of satellite joint scheduling to a certain extent; Li et al. [20] proposed a multi-objective binary-coded differential evolution algorithm (DEA), which can more efficiently obtain solutions with both excellent convergence and distribution; Chen et al. [21] proposed a multi-objective learning evolutionary algorithm specifically for solving the regional mapping problem in imaging satellite mission planning. However, genetic algorithms have problems such as being prone to local optima and parameter sensitivity. To this end, the adaptive genetic algorithm is proposed. Its core idea is to dynamically adjust the crossover probability and mutation probability according to the population evolution state during the algorithm iteration to balance the global search ability and local convergence ability of the algorithm, avoiding the “premature convergence” or “slow convergence” problems caused by fixed parameters in traditional genetic algorithms. For example, Yang et al. [22] improved the selection, crossover and mutation operators of traditional genetic algorithms for satellite network resource scheduling problems, and improved the problem of being prone to local optima through adaptive updating of mutation and crossover operators’ own probabilities.

Traditional adaptive methods mainly dynamically adjust crossover and mutation probabilities based on individual fitness or fitness differences to balance population diversity and local search capabilities. Among them, under the adjustment strategy based on individual fitness, when the individual fitness is lower than the average fitness, the crossover and mutation probabilities are increased to explore new solution spaces through gene recombination or mutation; when the individual fitness is higher than the average fitness, the crossover and mutation probabilities are reduced to avoid destroying existing high-quality gene combinations. Under the adjustment strategy based on fitness differences, when the fitness difference is smaller, the crossover and mutation probabilities are increased to improve population diversity; when the fitness difference is larger, the crossover and mutation probabilities are reduced to avoid destroying potential excellent genes when the population is relatively diverse. The above strategies can be implemented using linear functions, exponential functions, etc., according to actual tasks. Obviously, regardless of the strategy, the crossover and mutation probabilities change synchronously. However, crossover operators and mutation operators play different roles in genetic algorithms-crossover operators have better global search capabilities, while mutation operators have stronger local optimization capabilities. Only effective cooperation between the two can enable genetic algorithms to have balanced search capabilities that take both global and local into account. Therefore, it is necessary to further study the variation mode of adaptive genetic algorithms.

## 3. Construction of Distributed Task Model

### 3.1. Problem Description

In a space target surveillance constellation system composed of multiple visible light payload satellites, each satellite, in addition to basic observation and communication capabilities, also has a certain on-board computing power and the function of calculating the access time window of task targets. When a batch of information about the set of tasks to be observed arrives at any satellite in the constellation in the form of ground injection or on-board self-generation, this satellite acts as the master satellite, and other satellites (slave satellites) work together with it to allocate and plan the task set to achieve the best overall scheme. The task set contains a certain number of space targets and their observation time windows. The observation of any target requires occupying a period of working time of a space target surveillance satellite, and the execution time period must satisfy the visible time window and observation time window of the satellite for the target. Affected by dynamic disturbance factors such as satellite resources and tracking tasks, the task planning of space target surveillance constellations is dynamic. To improve the rapid response capability to emergencies, the task planning system of space target surveillance constellations generally adopts a re-planning strategy combining periodic re-planning and event-driven re-planning. It first triggers re-planning regularly according to a preset fixed period, and immediately re-plans when an unexpected event occurs. In each re-planning, the problem can be simplified to a static task planning problem. This paper mainly focuses on the static tasks of each planning to provide effective arc segment data for daily catalog management as the task goal.

### 3.2. Model Establishment

#### 3.2.1. Basic Assumptions

The task planning problem of space target surveillance constellations involves many factors. To exclude the interference of weakly relevant factors and highlight the research focus, and fully combine its application scenarios, the following basic assumptions are made in the modeling process:

(1) The observation duration of a single task is a fixed value;
(2) The problem of image recognition is not considered, that is, meeting the single continuous duration required for target observation is considered to meet the recognition requirements for the target;
(3) A satellite can only perform one task at a time and cannot be interrupted during execution;
(4) Each task performed by a satellite consumes a certain amount of time for attitude adjustment;
(5) Satellites have autonomous task planning capabilities and inter-satellite communication capabilities, can plan the received tasks, and do not consider the impact related to data transmission activities;
(6) The task planning time is in days. The observation capability of the satellite in a single task planning is represented by the daily available boot time of the satellite;
(7) Satellite storage and power resources are measured by the available boot time of the satellite. During the execution of observation tasks, the satellite not only needs to occupy a certain storage capacity, but also consumes a certain amount of power for activities such as switching on and off and attitude adjustment. Both quantities are linearly related to time. To simplify the setting of constraint conditions, this paper uses the available boot time constraint of the satellite to represent the satellite storage and power resource constraints.

#### 3.2.2. Parameter Definition

(1) Set of tasks

The task set is $T=\left\{T_{1},T_{2},\cdots ,T_{i},\cdots ,T_{NT}\right\},1\leq i\leq NT$, NT is the number of tasks to be planned. A single task is defined as $T_{i}=\langle ts_{i},tp_{i},td_{i},tw_{i}\rangle$, where $ts_{i}$ is the space target corresponding to the task $T_{i}$, $tp_{i}$ is the priority of the task $T_{i}$, $td_{i}$ is the minimum observation time required for the task $T_{i}$, $tw_{i}=\left\{tw_{j}^{i}\left|\exists j\in S\right.\right\}$ is the set of observation time windows for the satellite $S_{j}$ to perform the task $T_{i}$, $tw_{j}^{i}=\left\{tws_{j}^{i},twe_{j}^{i},twd_{j}^{i}\right\},i\in T,j\in S$, and $tws_{j}^{i},twe_{j}^{i},twd_{j}^{i}$, respectively, represent the start time, end time and duration of the satellite’s $S_{j}$ execution of the task $T_{i}$.

(2) Set of satellite resources

The satellite resource set is $S=\left\{S_{1},S_{2},\cdots ,S_{j},\cdots ,S_{NS}\right\},1\leq j\leq NS$, NS is the number of available satellites. Single satellite is defined as $S_{j}=\langle sd_{j},st_{j},sw_{j}^{i}\rangle$, where $sd_{j}$ is the available boot time of the satellite $S_{j}$, $st_{j}$ is the task conversion time of the satellite $S_{j}$, $sw_{j}^{i}=\left\{sw_{j1}^{i},\cdots ,sw_{jm}^{i},\cdots ,sw_{jn}^{i}\right\}$ is the set of visible time windows of the satellite $S_{j}$ for the task $T_{i}$, $sw_{jm}^{i}=\left\{sws_{jm}^{i},swe_{jm}^{i},swd_{jm}^{i}\right\}$ is the m-th visible time window set of the satellite $S_{j}$ for the task *T_i_*, and ${sws}_{\mathrm{jm}}^{i},{swe}_{\mathrm{jm}}^{i},{swd}_{\mathrm{jm}}^{i}$, respectively, represent the start time, end time and duration of the visible time window.

(3) Decision variables

The task decision variable represents whether the task is observed, defined as $x_{j}^{i}$; when the satellite $S_{j}$ executes the task $T_{i}$, $x_{j}^{i}=1$; otherwise, $x_{j}^{i}=0$. At the same time, two auxiliary decision variables $y_{jm}^{i}$ and $z_{j}^{ab}$ are defined. When the satellite $S_{j}$ executes the task $T_{i}$ in the m-th visible time window, $y_{jm}^{i}=1$; otherwise, $y_{jm}^{i}=0$. When, on the satellite $S_{j}$, task $T_{a}$ is the predecessor of task $T_{b}$, $z_{j}^{ab}=1$; otherwise, $z_{j}^{ab}=0$.

#### 3.2.3. Distributed Task Planning Model

##### Slave Satellite Task Planning Model

(1) Optimization Objectives

As the main body of the task planning problem in this paper, the slave satellite has multiple optimization objectives.

Sub-objective 1: Maximize task yield. Herein, the priority of the task target is equated to the task benefit value.

(1) $$f_{1}=\max\left[(\sum_{j=1}^{NS}x_{j}^{i}\cdot tp_{i})/\sum_{i=1}^{NT}tp_{i}\right]$$

Sub-objective 2: Maximize task completion rate.

(2) $$f_{2}=\max\left[\left(\sum_{i=1}^{NT}x_{j}^{i}\right)/NT\right]$$

Sub-objective 3: Maximize resource utilization. Maximizing resource utilization refers to achieving the maximum task benefit output with the minimum resource input through rational allocation and efficient use of various resources. Therefore, this paper uses minimizing the occupation of satellite available boot time to represent maximizing resource utilization.

(3) $$f_{3}=\min\left\{\left[\sum_{i=1}^{NT}x_{j}^{i}\cdot (twd_{j}^{i}+st_{j})\right]/sd_{j}\right\}$$

The three optimization sub-objectives of the slave satellite task planning model are designed into a single overall optimization objective using the weighted sum method, which is specifically expressed as follows:

(4) $$f_{s}=\alpha f_{1}+\beta f_{2}+\lambda f_{3}$$

where α,β,λ are the weight indicators of the three sub-objectives, each ranging from −1 to 1, and α+β+λ=1. The value of the weight reflects the importance of the sub-objective in the overall optimization objective, and different weight combination schemes can be set according to different task requirements.

(2) Constraints

Constraint 1: Satellite task transition time constraint. After a satellite completes one task, it needs to adjust its attitude before proceeding to the next task, that is, when a satellite needs to execute task $t_{a}$ and task $t_{b}$ successively, the time interval between the two tasks must be no less than the satellite’s task transition time.

(5) $$C_{1}:tws_{j}^{b}-(twe_{j}^{a}+st_{j})\geq 0,\forall j\in S,x_{j}^{a}=x_{j}^{b}=1,z_{j}^{ab}=1$$

Constraint 2: Visible time window constraint. The actual observation window for a satellite to execute a task must be contained within the satellite’s visible time window for the task, and the observation duration must meet the minimum observation duration required for the task.

(6) $$C_{2}:tws_{j}^{i}\leq sw_{jm}^{i},twd_{j}^{i}\geq td_{i},\forall tw_{j}^{i}\in tw_{i},y_{jm}^{i}=1$$

Constraint 3: Available boot time constraint. The total time occupied by all activities of a satellite within the task planning cycle must not exceed the maximum boot time of the satellite.

(7) $$C_{3}:\sum_{i=1}^{NS}x_{j}^{i}\cdot (twd_{j}^{i}+st_{j})\leq sd_{j},\forall j\in S$$

Constraint 4: Task execution frequency constraint. Each satellite can execute at most one task within a visible time window.

(8) $$C_{4}:\sum_{i=1}^{NT}x_{j}^{i}\cdot y_{jm}^{i}\leq 1,\forall j\in S,\forall m\in n$$

##### Master Satellite Task Decision Model

(1) Optimization Objectives

To obtain the global optimal solution, the master satellite will make targeted adjustments to the task planning schemes of each slave satellite based on the following sub-objectives. Among them, the specific adjustment methods of the master satellite will be detailed in Section 4.3, while this section focuses on introducing the adjustment principles of the master satellite.

Sub-objective 4: Maximize the total task yield. Compared with the slave satellite task planning, which mainly considers maximizing the yield of tasks executed by a single satellite and takes the task priority as the task benefit value, the master satellite task decision-making comprehensively considers the task and satellite execution efficiency. In view of the high timeliness requirement of space target surveillance tasks, this paper proposes a relative task benefit value that combines task priority and observation time.

Taking task $T_{i}$ as an example, its priority is $tp_{i}$, multiple slave satellites can observe it and determine its observation time windows, and the set of their respective observation start times is $tws^{i}=\left\{tws_{j}^{i}\left|x_{j}^{i}=1\right.\right\}$, where $tws_{m}^{i}$ is the latest start time. The relative task benefit value is expressed as:

(9) $$P_{j}^{i}=tp_{i}\cdot \frac{tws_{m}^{i}}{tws_{j}^{i}}$$

Then, the optimization objective is expressed as:

(10) $$f_{4}=\max\left(\sum_{i=1}^{NT}\sum_{j=1}^{NS}P_{j}^{i}\cdot x_{j}^{i}\right)$$

Sub-objective 5: Maximize resource load balancing, that is, to make the load of each satellite assigned tasks as balanced as possible, and make full use of multi-satellite resources to execute tasks collaboratively. To evaluate the resource load balancing status of multiple satellites, this study introduces the Standard Deviation (std)—a statistical measure commonly used to describe the degree of dispersion of data relative to the mean. Taking the ratio of effective operating time to available boot time of a single satellite as the calculation unit, this measure enables the determination of the satellites’ resource load balancing status by analyzing the degree of dispersion of the aforementioned ratio data across multiple satellites: if there is a high degree of data dispersion, it indicates a significant imbalance in resource load among the multiple satellites; otherwise, it means the resource load of the multiple satellites is in a relatively balanced state.

(11) $$f_{5}=\max\left\{1-std\left[(\sum_{i=1}^{NT}x_{1}^{i}\cdot twd_{1}^{i})/sd_{1},(\sum_{i=1}^{NT}x_{2}^{i}\cdot twd_{2}^{i})/sd_{2},\cdots ,(\sum_{i=1}^{NT}x_{NS}^{i}\cdot twd_{NS}^{i})/sd_{NS}\right]\right\}$$

Similarly, this paper uses the weighted sum method to convert multiple optimization objectives into a single overall optimization objective, which is specifically expressed as follows:

(12) $$f_{z}=af_{4}+bf_{5}$$

where a,b are the weight indicators of the two sub-objectives, each ranging from −1 to 1, and a+b=1. The value of the weight reflects the importance of the sub-objective in the overall optimization objective, and different weight combination schemes can be set according to different task requirements.

(2) Constraints

Constraint 5: Task execution frequency constraint. On the one hand, space target surveillance tasks oriented to space target arc tracking require multiple arc data, so multiple task executions are needed. On the other hand, excessive observation of a certain target to some extent wastes satellite observation resources. Therefore, it is necessary to constrain the number of task executions. The maximum number of executions k is related to the length of the entire observation cycle, task importance, etc.

(13) $$C_{5}:2\leq \sum_{j=1}^{NS}x_{j}^{i}\leq k,\forall i\in T$$

#### 3.2.4. Space Target Surveillance Constellation Distributed Task Planning Model

In summary, based on the idea that each slave satellite provides a local optimal solution and the master satellite comprehensively compares to obtain a solution close to the global optimal solution, a distributed task planning model for space target surveillance constellations is constructed:

(14) $$\left\{\begin{array}{l} \max f_{s}=\alpha \left[(\sum_{j=1}^{NS}x_{j}^{i}\cdot tp_{i})/\sum_{i=1}^{NT}tp_{i}\right]+\beta \left[\left(\sum_{i=1}^{NT}x_{j}^{i}\right)/NT\right]-\lambda \left\{\left[\sum_{i=1}^{NT}x_{j}^{i}\cdot (twd_{j}^{i}+st_{j})\right]/sd_{j}\right\}\\ s.t.\left\{\begin{array}{l} tws_{j}^{b}-(twe_{j}^{a}+st_{j})\geq 0,\forall j\in S,x_{j}^{a}=x_{j}^{b}=1,z_{j}^{ab}=1\\ tws_{j}^{i}\leq sw_{jm}^{i},twd_{j}^{i}\geq td_{i},\forall tw_{j}^{i}\in tw_{i},y_{jm}^{i}=1\\ \sum_{i=1}^{NS}x_{j}^{i}\cdot (twd_{j}^{i}+st_{j})\leq sd_{j},\forall j\in S\\ \sum_{i=1}^{NT}x_{j}^{i}\cdot y_{jm}^{i}\leq 1,\forall j\in S,\forall m\in n \end{array}\right.\\ \max f_{z}=a\left(\sum_{i=1}^{NT}\sum_{j=1}^{NS}P_{j}^{i}\cdot x_{j}^{i}\right)+b\left\{1-std\left[(\sum_{i=1}^{NT}x_{1}^{i}\cdot twd_{1}^{i})/sd_{1},(\sum_{i=1}^{NT}x_{2}^{i}\cdot twd_{2}^{i})/sd_{2},\cdots ,(\sum_{i=1}^{NT}x_{NS}^{i}\cdot twd_{NS}^{i})/sd_{NS}\right]\right\}\\ s.t.2\leq \sum_{j=1}^{NS}x_{j}^{i}\leq k,\forall i\in T \end{array}\right.$$

The finally output specific implementation model of space target surveillance constellation task planning can not only achieve the optimal task total benefit and task completion rate as much as possible, but also achieve relatively balanced resource load and maximum resource utilization as much as possible.

## 4. Distributed Task Planning and Scheduling Algorithm (DTP&SA) Based on the Adaptive Genetic Algorithm

**Figure 3:**
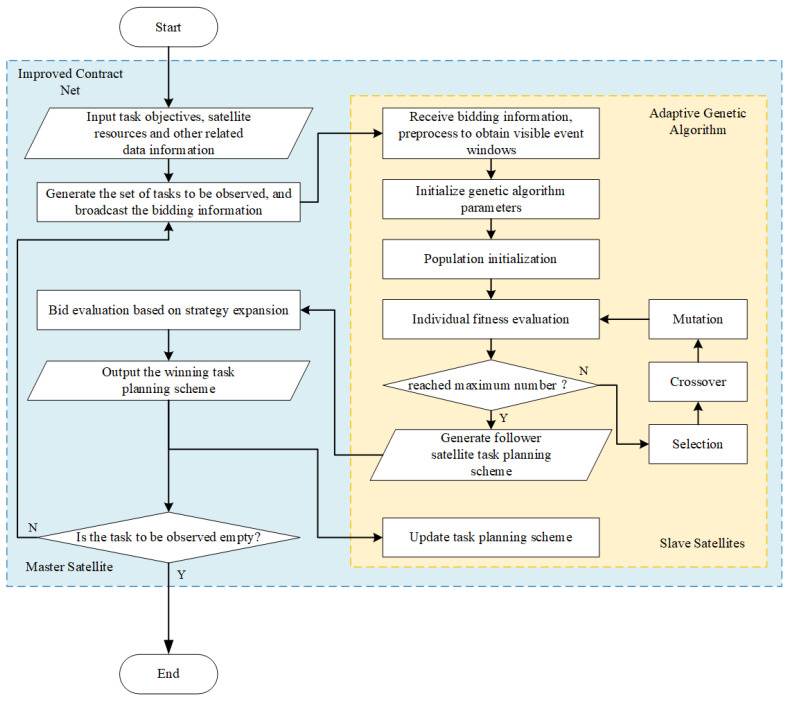
Flowchart of Distributed Task Planning and Scheduling Method Based on Adaptive Genetic Algorithm.

To address the distributed task planning model mentioned above, this paper designs a distributed task planning and scheduling algorithm based on the adaptive genetic algorithm, which combines the contract net algorithm and the adaptive genetic algorithm, the algorithm flow is shown in Figure 3. This section describes the algorithm in detail according to the three stages of the contract net algorithm: bidding, tendering, and winning the bid.

### 4.1. Tendering Stage

To address the problems of high communication cost and low negotiation efficiency of the traditional contract net algorithm, this method adopts a centralized bidding strategy in the tendering stage, that is, the master satellite conducts centralized bidding for all space target surveillance tasks to be planned in each round of bidding, reducing the number of negotiations in the bidding process and lowering inter-satellite communication volume.

### 4.2. Bidding Stage

When each slave satellite generates its own bidding scheme, to improve the utilization rate of satellite resources, this paper employs the genetic algorithm (GA) instead of the “insertion” strategy to generate the bidding scheme. Meanwhile, to further improve the performance of the genetic algorithm, the algorithm’s genetic operators are optimized and a dynamic population strategy is introduced. In addition, to further improve the efficiency of the contract net algorithm and conserve satellite communication resources, before bidding, each slave satellite independently judges whether the new task planning yield is higher than the original task planning scheme. If yes, it bids; otherwise, it does not participate in tendering. The solution steps for generating the slave satellite task planning scheme using the adaptive genetic algorithm are as follows:

(1) Set the genetic generation counter GEN = 1.
(2) Chromosome coding

**Figure 4:**
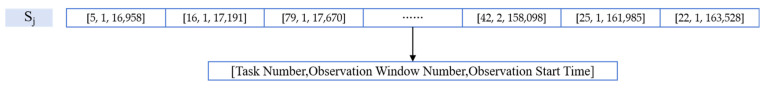
Schematic Diagram of Coding.

This paper adopts integer coding to solve the slave satellite task planning problem. For a slave satellite with M imaging tasks, each task has multiple visible time windows. The coding of a feasible solution is shown in Figure 4. The entire coding consists of different gene positions, and a specific gene position contains three parts: the imaging task number, the assigned observation window number, and the observation start time.

(3) Initial population generation

A set of chromosomes is randomly generated as the initial population for task planning, with the population size set to 20.

(4) Fitness evaluation

The is used $f_{s}$ as the fitness function for calculation to measure the quality of the corresponding solution.

(5) Selection

This paper improves the selection operator using the roulette wheel selection method and the elitist preservation strategy, through a collaborative mechanism of “probability-driven-deterministic guarantee”. On the one hand, in the early stage of evolution, the high-probability exploration ability of the roulette wheel promotes the population to quickly expand the solution space. On the other hand, in the middle and late stages of evolution, the deterministic preservation mechanism of the elitist strategy ensures that high-quality solutions are not destroyed by the randomness of crossover and mutation.

The roulette wheel method is a commonly used selection operator in genetic algorithms, whose core principle is to determine the probability of an individual being selected according to the proportion of the individual’s fitness to the total fitness of the population. Its advantages are simple operation and conformity to the theory of probabilistic evolution, but it has sampling errors, which may cause high-fitness individuals not to be selected due to random fluctuations. At the same time, the “Matthew effect” of the roulette wheel selection method (high-fitness individuals have a higher probability of being selected) may lead to rapid genetic homogenization of the population and falling into local optima.

The elitist preservation strategy refers to directly retaining some of the best individuals in the current generation into the next generation population, that is, copying a certain number of individuals with the highest fitness values in the current population, which are directly added to the next generation population without any modification by genetic operators such as crossover and mutation. This strategy can effectively ensure that the current optimal individuals in each generation will not be lost due to subsequent crossover and mutation operations, which is conducive to faster convergence of the algorithm.

(6) Crossover and mutation

To enable the algorithm to converge to the global optimal solution more efficiently and achieve a balance between global search and local optimization, this paper adopts an adaptive method to determine the crossover and mutation probabilities according to the fitness value of the current individual. Different from the synchronous change in crossover and mutation operators in conventional adaptive methods, to further utilize the global search ability of the crossover operator and the local optimization ability of the mutation operator, this paper refers to the calculation method in reference [23] and adds consideration of individual fitness on this basis, combining individual fitness with population fitness difference, and using inverse trigonometric functions to adaptively improve the crossover and mutation operators, so that the two change inversely. The expressions are as follows:

(15) $$P_{c}=\left\{\begin{array}{l} k_{1}\left(1-\frac{\arcsin\left(\frac{\bar{F}}{F_{\max}}\right)}{\pi /2}\right),f^{\prime }<\bar{F}\\ k_{1}\left(1-\frac{\arcsin\left(\frac{\bar{F}}{F_{\max}}\right)}{\pi /2}\right)\cdot \left(1-\frac{f^{\prime }-\bar{F}}{F_{\max}-\bar{F}}\right),f^{\prime }\geq \bar{F} \end{array}\right.$$

(16) $$P_{m}=\left\{\begin{array}{l} k_{2}\left(\frac{\arcsin\left(\frac{\bar{F}}{F_{\max}}\right)}{\pi /2}\right),f^{\prime }<\bar{F}\\ k_{2}\left(\frac{\arcsin\left(\frac{\bar{F}}{F_{\max}}\right)}{\pi /2}\right)\cdot \left(1+\frac{f^{\prime }-\bar{F}}{F_{\max}-\bar{F}}\right),f^{\prime }>\bar{F} \end{array}\right.$$

In the formulas, $F_{\max}$ is the maximum fitness value among all individuals in the population, $\bar{F}$ is the average fitness value of all individuals in the population, $f^{\prime }$ is the individual fitness value, $k_{1}$ and $k_{2}$ are constants, and $0<k_{1},k_{2}<1$, in addition, it is specified that $P_{c}\in \left[P_{c}^{\min},P_{c}^{\max}\right],P_{m}\in \left[P_{m}^{\min},P_{m}^{\max}\right]$.

**Figure 5:**
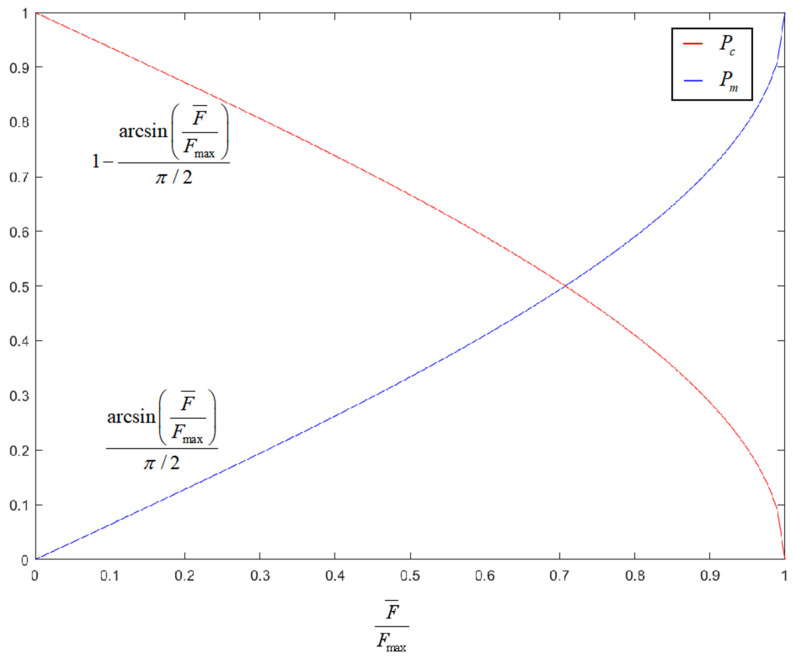
Trend chart of crossover and mutation operators changing with fitness difference.

In terms of the overall fitness difference in the population, as the difference between the maximum fitness of individuals in the population and the overall average fitness gradually decreases, the individual fitness in the population changes from relatively scattered to relatively concentrated, and the population diversity gradually decreases. The $P_{c}$ will be adaptively reduced, while the $P_{m}$ will be adaptively increased. In the early stage, the crossover probability is high, which makes it easy to generate high-quality individuals, and the mutation probability is low, which reduces the possibility of potential excellent genes being destroyed, accelerating the convergence speed; in the later stage, the crossover probability is low, which avoids invalid crossover and reduces the waste of computing resources, and the mutation probability is high, which helps to jump out of local optimal traps and maintain population diversity, to a certain extent making up for the shortcoming that the roulette wheel is prone to falling into local optima. The overall change is shown in Figure 5.

**Figure 6:**
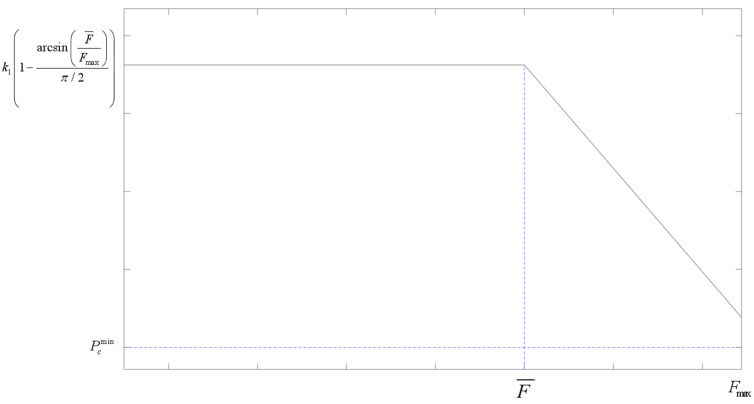
Adaptive crossover operator.

**Figure 7:**
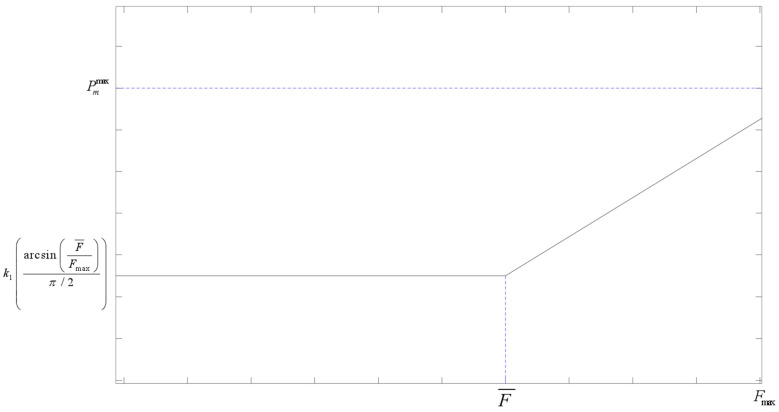
Adaptive mutation operator.

In terms of individual fitness, in a single iteration, when the individual’s fitness is lower than the average fitness of the population, it indicates that the individual is far from the optimal solution, and the global search ability of the algorithm should be reasonably improved, so the crossover probability is higher and the mutation probability is lower; when the individual’s fitness is higher than the average fitness of the population, the local optimization ability of the algorithm should be improved, so the mutation probability is increased and the crossover probability is decreased. At the same time, due to the application of the elitist preservation strategy, the current optimal individual of each generation will not be lost due to crossover and mutation operations. The adjustment curves are shown in Figure 6 and Figure 7.

(7) Solving the optimal solution

Substitute each obtained chromosome into the objective function to solve the fitness value. For the weight values of the objective function, determination is performed via multiple sets of controlled experiments in this study. Specifically, the value interval of each weight is first defined by setting its maximum and minimum values, followed by gradient adjustment of weights with a fixed step size while simulation results of representative samples are calculated. Through comparative verification of multiple sets of results, it is found that the weight combination (0.5, 1, −0.5) can achieve superior simulation performance.

When the evolution results of consecutive generations do not change significantly or the maximum number of genetic generations is reached, the chromosome with the largest current fitness value is considered as the best slave satellite task planning scheme, and the algorithm ends; otherwise, proceed to the next iteration.

Before each iteration starts, to increase population diversity, a dynamic population strategy is introduced. First, new d individuals are generated with probability μ and added to the population to participate in the selection, crossover, and mutation processes. Among them, d is smaller than the initial population size, 0<μ<1; second, after the genetic operation, new individuals will be generated. When the fitness of the new individual is higher than that of the parent individual, it is added to the new generation population; otherwise, the parent individual is retained and directly enters the new generation population.

### 4.3. Winning the Bid Stage

In the bid evaluation and winning stage, this paper divides tasks into non-repetitive tasks and repetitive tasks according to the number of times they are planned. For non-repetitive tasks, a centralized winning strategy is adopted, that is, all non-repetitive tasks are directly won by the tendering slave satellites, which saves the time and computing resources consumed in evaluating these tasks on the basis of making full use of slave satellite computing resources.

For repetitive tasks, a bid evaluation strategy based on conflict resolution is adopted. First, the task $T_{i}$ conflict degree is defined as:

(17) $$\gamma_{i}=\frac{N_{i}}{\left|S\right|}\cdot tp_{i}$$

where $N_{i}$ represents the number of slave satellites whose bidding schemes include repeatedly planned tasks $T_{i}$, $\left|S\right|$ represents the number of bidding schemes, and $tp_{i}$ represents the priority of the task. Sort the repetitive tasks according to the conflict degree from high to low, and evaluate the bids for repetitive tasks. At the same time, this paper proposes two conflict resolution principles: (1) priority is given to those with higher task yield; (2) if the task yields are the same, priority is given to those with fewer tasks undertaken.

The distributed task planning model for space target surveillance constellations has been constructed above, and a distributed task planning and scheduling method combining the adaptive genetic algorithm and the contract net algorithm has been designed, specifying the basic assumptions, parameter definitions, optimization objectives, constraint conditions of the model, and specific strategies for each stage of the algorithm. To verify the effectiveness and superiority of the proposed model and method, the following will conduct detailed verification through simulation experiments and analyze the results.

## 5. Simulation Verification and Result Analysis

### 5.1. Simulation Scenario

The space target surveillance mission studied in this paper is to provide tracking arc data that meets the daily cataloging needs of space targets. With reference to the mainstream orbital types of operational space target surveillance satellites, the visible light payload characteristics of existing satellites of the same type, and the space target surveillance constellations proposed in Reference [24], this study employs a low Earth orbit (LEO) satellite constellation consisting of two Walker sub-constellations for simulations. The two sub-constellations have orbital altitudes of 550 km and 700 km, respectively, and both adopt a configuration of 6/1/0. Herein, the configuration parameter 6/1/0 is defined as follows: 6 represents the total number of satellites (T) in each sub-constellation; 1 denotes the number of orbital planes (P) in each sub-constellation; and 0 indicates the phasing factor (F) of each sub-constellation. The 12 satellites of the entire constellation (comprising the two sub-constellations) are numbered 1 to 12, with Satellites 1–6 belonging to the 550 km sub-constellation and Satellites 7–12 to the 700 km sub-constellation; their specific parameters are presented in Table 1 and Table 2.

In addition to the constellation configuration, the selection strategy of the master satellite is specified as follows: In practical applications, the master satellite is selected in accordance with the principle described in Section 3.1, i.e., the satellite closest to the ground station, which facilitates the master satellite’s reception of observation task instructions. In the simulation of this study, Satellite 1 is designated as the master satellite, which undertakes the task decision-making function.

To verify the feasibility and applicability of the algorithm proposed in this paper, referring to reference [24], low-orbit targets are classified into three categories according to their orbital altitudes, namely those with an orbital altitude below 450 km, those between 450 km and 600 km, and those above 600 km. 100 targets are randomly selected from each category as the objects of the space target surveillance mission. The target priority is randomly generated in [1,10], with larger values indicating higher priority. To reduce the complexity of the simulation, the orbital motion models of both the surveillance satellite and the target in this paper all adopt the J2 orbital perturbation. The arc validity constraints refer to reference [25], as shown in Table 3.

### 5.2. Simulation Results

Under the above simulation scenario, the distributed space target surveillance constellation task planning method based on the adaptive genetic algorithm proposed in this paper is simulated.

After multiple cycles of bidding, tendering, evaluation, and award, the number of satellite observation tasks under different orbital altitude categories as well as the overall task completion rate are shown in Table 4. Overall, in the observation missions for targets at different orbital altitudes, the task completion rate basically reaches 80%, achieving good results.

**Figure 8:**
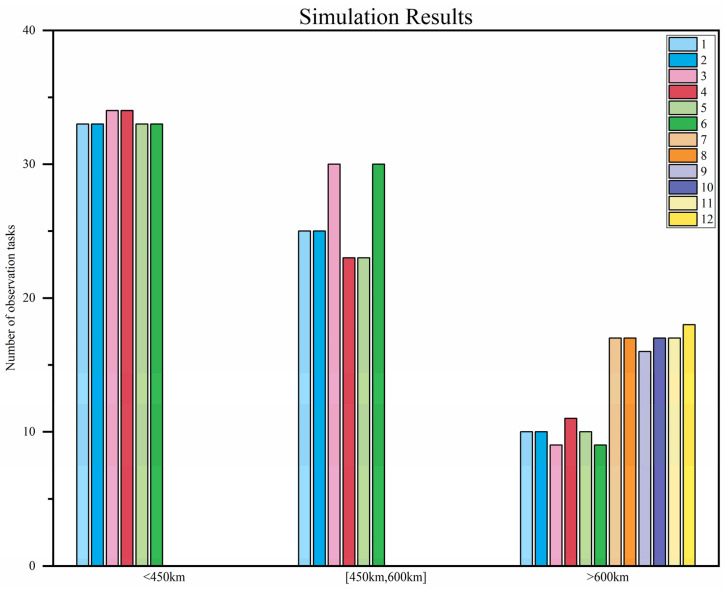
Simulation results.

Figure 8 shows the number of tasks undertaken by each satellite for targets at different orbital altitudes.

Overall, space target surveillance satellites at the same orbital altitude undertake a similar number of tasks, with relatively balanced loads. However, significant differences emerge in the performance of surveillance satellites at different orbital altitudes when dealing with targets at varying orbital heights: when the target orbital altitude is below 600 km, satellites at 700 km orbital altitude take on zero tasks, while those at 550 km orbital altitude assume most of the observation tasks—with a 100% task completion rate for targets below 450 km. When the target orbital altitude exceeds 600 km, satellites at 700 km orbital altitude undertake more observation tasks than those at 550 km orbital altitude.

The main reason for this phenomenon lies in the configuration design of the constellation. The space target surveillance constellation and sensor pointing adopted in this paper all refer to reference [24]. In this constellation, satellites at different orbital altitudes have different visible event windows for space targets at different orbital altitudes, which leads to differences in the number of tasks undertaken by each space target surveillance satellite during task planning.

The above content has provided an overall overview of the application of the distributed task planning algorithm based on the adaptive genetic algorithm proposed in this paper to this problem. Next, the operation of the algorithm will be elaborated on and analyzed in detail from the two dimensions of the master satellite and the slave satellite.

#### 5.2.1. Master Satellite Task Decision Results

**Figure 9:**
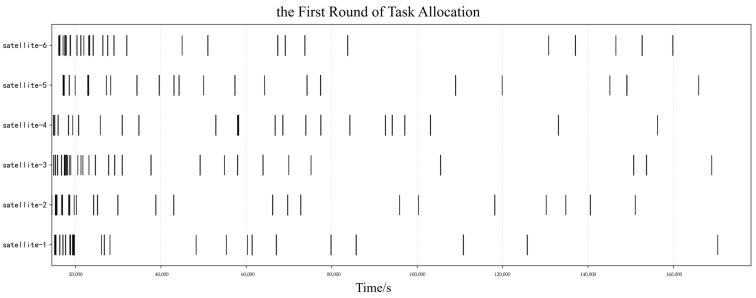
Gantt Chart of the First Round of Task Allocation: Target Orbital Altitudes Between 450 and 600 km.

**Figure 10:**
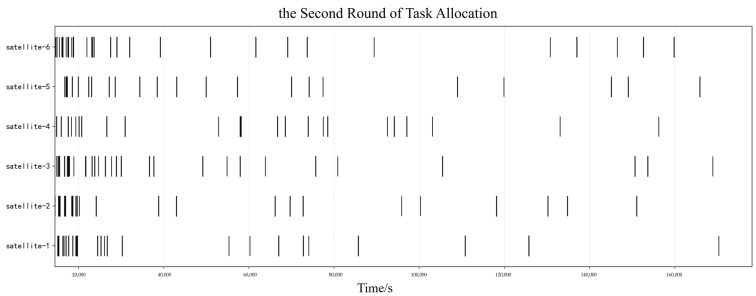
Gantt Chart of the Second Round of Task Allocation: Target Orbital Altitudes Between 450 and 600 km.

Taking the tasks with target orbital altitudes between 450 and 600 km as an example, their task allocation Gantt charts are shown in Figure 9 and Figure 10. During the simulation process, this study set the maximum number of bidding and tendering iterations to 20. The result is deemed to have converged when the assigned task IDs are completely consistent across two consecutive iterations, and the algorithm terminates accordingly. Specifically, for this type of task, after two rounds of slave satellites’ planning and master satellite’s decision-making, the planning scheme successfully converges. This is reflected in the fact that both the unallocated task sets and allocated task sets of the slave satellites remain stable and unchanged.

**Figure 11:**
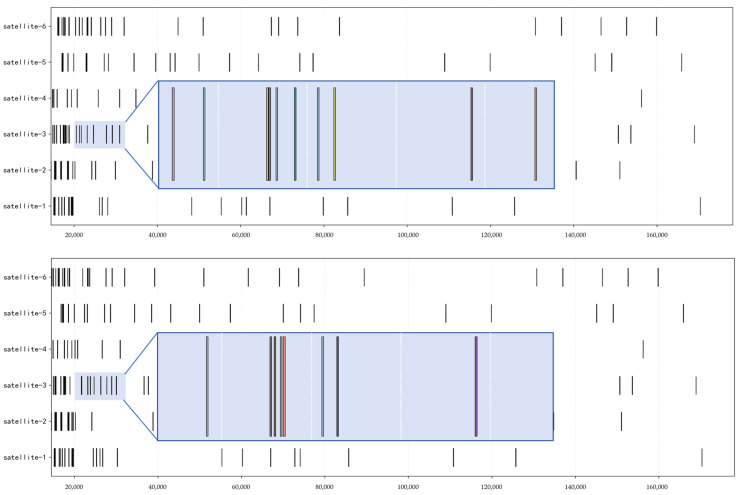
Comparison of Two-Round Task Planning Schemes: Taking Partial Planning Tasks of Satellite 3 as an Example.

A comparison of Figure 9 and Figure 10 reveals that the time windows of some tasks have changed significantly, such as those for Satellite 3—a phenomenon shown in Figure 11. This is because after the first round of master satellite decision-making, some tasks of the slave satellites were eliminated, and during the second round of slave satellite planning, the task windows were adjusted independently to obtain a higher fitness value. From the perspective of the time axis, the distribution is relatively dense at earlier times than at later times, which is in line with the requirement of pursuing high timeliness.

#### 5.2.2. Slave Satellite Task Planning Results

**Figure 12:**
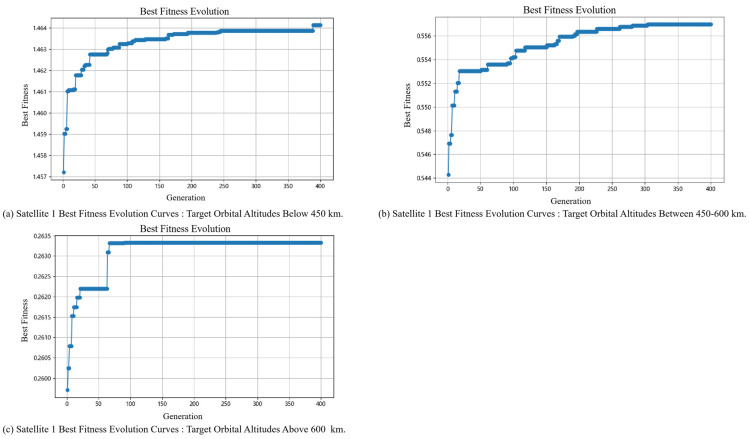
Satellite 1 Best Fitness Evolution Curves for Different Task Sets.

**Figure 13:**
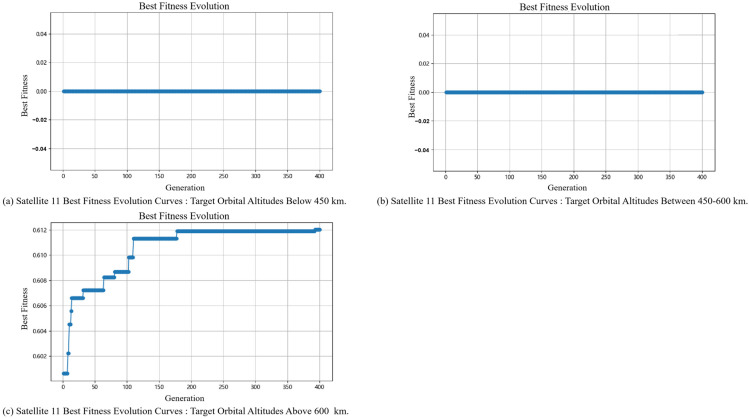
Satellite 11 Best Fitness Evolution Curves for Different Task Sets.

Regarding the application of the algorithm proposed in this paper in the process of slave satellite task planning, the analysis focuses on the role of the adaptive genetic algorithm in slave satellite task planning under different problem scales. Satellite 1 and Satellite 11 are selected as representatives of different orbital altitudes, among which Satellite 1 and Satellite 11 represent constellations with orbital altitudes of 550 km and 700 km, respectively, and the relevant results are shown in Figure 12 and Figure 13.

Figure 12 and Figure 13 show the iterative convergence process of the algorithm under different tasks. In the figures, the abscissa represents the number of iterations, and the ordinate comprehensively considers task benefits, task completion rate, and resource utilization. In general, when targeting objects at different orbital altitudes, the adaptive genetic algorithm proposed in this paper can finally achieve convergence. Regarding the initial fitness value, since visibility and validity issues have been considered when generating the initial population in this paper, the search range is narrowed to a certain extent. Therefore, regardless of the task target, the gap between the initial fitness value and the final fitness value of Satellite 1 and Satellite 11 is relatively small.

Similar to the analysis of the final results presented above, as affected by the constellation’s inherent configuration, space target surveillance satellites operating at orbital altitudes of 550 km and 700 km exhibit obvious sensitivity to targets at different orbital altitudes—and their fitness values show a strong correlation with the orbital altitude of targets. For Figure 12, compared with Panels (a) and (b), the fitness values in Panel (c) decrease significantly. This indicates that space target surveillance satellites at 550 km exhibit worse tracking performance for targets with an orbital altitude exceeding 600 km; instead, these 550 km-altitude satellites are more suitable for tracking targets below 600 km. For Figure 13, the fitness values in both Panels (a) and (b) are 0, while those in Panel (c) are generally higher. This result demonstrates that, by contrast, space target surveillance satellites at 700 km achieve better tracking performance for targets with an orbital altitude greater than 600 km. Therefore, by further analyzing future mission requirements and integrating the current task planning results, the constellation configuration can be adjusted in a targeted manner.

In summary, the simulation results show that the distributed space target surveillance constellation task planning method based on the adaptive genetic algorithm proposed in this paper can meet the arc segment requirements of around 80% of space target cataloging, and to a certain extent, can achieve relatively balanced satellite load and high timeliness of tasks.

#### 5.2.3. Algorithm Comparison

In this section, a comparative experiment is conducted between the distributed task planning and scheduling algorithm (DTP&SA) based on the adaptive genetic algorithm (AGA) proposed in this paper, and the unimproved distributed task planning and scheduling algorithm based on the genetic algorithm (GA) (i.e., the unimproved contract net task planning and scheduling algorithm). The two algorithms share the same overall framework, with core differences lying in the bidding phase and the bid evaluation phase:

- In the bidding phase, the subtask planning algorithm of the unimproved algorithm adopts the classical GA, specifically using the roulette wheel selection algorithm, and sets fixed crossover probability (0.6) and mutation probability (0.1);
- In the bid evaluation phase, the unimproved algorithm does not adopt the centralized winning strategy, but instead uses the method of evaluating bids one by one.

Taking the target samples with an orbital altitude ranging from 450 km to 600 km as an example, the two algorithms are run and tested separately, and finally the operation results are obtained (see Table 5). For clarity, satellites not assigned tasks are not presented in the table.

From the above experimental results, the improved algorithm has more significant advantages over the standard algorithm (i.e., the unimproved GA-based algorithm) in terms of both task completion rate and computational efficiency. By calculating the variance of the number of tasks undertaken by the six satellites under the two algorithms, the balance of resource load can be reflected to a certain extent: the variance of the number of tasks undertaken by the six satellites under the improved algorithm is 2.57, while that under the standard algorithm is 4.07. It is evident that the planning scheme generated by the improved algorithm is more balanced in resource load allocation.

The above simulation experiments effectively verify that, for the task planning problem of the space target surveillance constellation, the distributed task planning and scheduling algorithm (DTP&SA) based on the adaptive genetic algorithm (AGA) proposed in this paper exhibits good applicability and efficiency.

## 6. Conclusions

Based on a given space target surveillance constellation, this paper conducts research on the planning method for space target surveillance tasks. The proposed distributed task planning method for space target surveillance constellations based on the adaptive genetic algorithm can, to a certain extent, realize a round of static planning tasks, providing cataloging and orbit determination arcs for approximately 80% of low-orbit space targets, thus demonstrating feasibility.

The task planning problem of space target surveillance constellations is not only related to the task planning method, but its final results are also affected by the configuration of the space target surveillance constellation. This study finds that space target surveillance satellites at different orbital altitudes have different sensitivities to space target tasks at different orbital altitudes. Therefore, further optimization of the space target surveillance constellation configuration can be carried out based on the task planning results of this paper, or a suitable constellation configuration can be selected according to target characteristics.

In addition, the dynamics of space target surveillance tasks are their most prominent feature. Hence, future research will focus on dynamically adjusting task planning schemes based on event-driven mechanisms. Moreover, in addition to providing arc data, space target surveillance constellations also undertake tasks such as staring tracking, which will become the next research direction in the field of task planning.

## Figures and Tables

**Table 1:** Space target surveillance constellation configuration.

Orbital Inclination (°)	Orbital Altitude (km)	T	P	F	Orbital Type
97.6	550	6	1	0	Sun-synchronous dawn-dusk orbit
98.2	700	6	1	0	Sun-synchronous dawn-dusk orbit

**Table 2:** Optical payload attributes.

Payload	Distance (km)	Imaging Resolution	Aperture (mm)	Lateral Relative Velocity (km/s)	Field of View (°)	Angular Resolution (″)
CCD/CMOS	300	4096 × 4096	120	3	9 × 9	8.8

**Table 3:** Requirements for the minimum observation arc length of space target initial orbit determination by space-based surveillance.

Target Type	Minimum Observation Arc Length (Single Satellite) (s)	Minimum Observation Arc Length (Double Satellites) (s)
LEO	400	40
GEO	1700	450

**Table 4:** Simulation results.

Target Orbital Altitude	Satellite Number	Number of Observation Tasks	Proportion of Observed Tasks/%
<450 km	1	33	100
	2	33	
	3	34	
	4	34	
	5	33	
	6	33	
	7	0	
	8	0	
	9	0	
	10	0	
	11	0	
	12	0	
[450 km,600 km]	1	25	78
	2	25	
	3	30	
	4	23	
	5	23	
	6	30	
	7	0	
	8	0	
	9	0	
	10	0	
	11	0	
	12	0	
>600 km	1	10	80.5
	2	10	
	3	9	
	4	11	
	5	10	
	6	9	
	7	17	
	8	17	
	9	16	
	10	17	
	11	17	
	12	18	

**Table 5:** Experimental Results of Algorithm Comparison.

Target Orbital Altitude	Algorithm	Satellite	Number of Observation Tasks	Proportion of Observed Tasks/%	Time/s
450–600 km	DTP&SA based on AGA	1	25	78	36.29
		2	25		
		3	30		
		4	23		
		5	23		
		6	30		
	the unimproved GA-based algorithm	1	23	67.5	39.4
		2	23		
		3	30		
		4	23		
		5	17		
		6	19		

## Data Availability

The data presented in this study are available on request from the corresponding author. The data are not publicly available due to private reason.
